# 

**Published:** 1875-01

**Authors:** 


### OUR EXCHANGE LIST.

| | Price per Year. | With this Journal. |
|---|---|---|
| Atlanta Daily Herald | $10 | $12 00 |
| Atlanta Daily Commonwealth | 6 | 8 50 |
| Rome Daily Commercial | 6 | 8 50 |
| The Galaxy | 4 | 6 50 |
| Popular Science Monthly | 5 | 7 50 |
| Medical Record | 5 | 7 50 |
| Rich. and Lou. Medical Journal | 5 | 7 50 |
| Medical and Surgical Reporter | 5 | 7 50 |
| London Lancet | 5 | 7 50 |
| American Journal Medical Sciences | 5 | 7 50 |
| Obstetrical Journal Great Britain and Ireland | 5 | 7 50 |
| New Orleans Medical and Surgical Journal | 5 | 7 50 |
| Philadelphia Medical Times | 5 | 7 50 |
| Chicago Medical Journal | 4 | 6 50 |
| Journal Nervous and Mental Diseases | 4 | 6 50 |
| New York Medical Journal | 4 | 6 50 |
| Boston Medical and Surgical Journal | 4 | 6 50 |
| American Practitioner | 3 | 5 50 |
| The Clinic | 3 | 5 50 |
| Archives of Electrology and Neurology | 3 | 5 50 |
| Lancet and Observer | 3 | 5 50 |
| Medical Examiner | 3 | 5 50 |
| Pacific Medical and Surgical Journal | 3 | 5 50 |
| The Western Lancet | 3 | 5 50 |
| St. Louis Medical and Surgical Journal | 3 | 5 50 |
| Nashville Journal Medicine and Surgery | 3 | 5 50 |
| Detroit Review Medicine and Pharmacy | 3 | 5 50 |
| The Sanitarian | 3 | 5 50 |
| Americal Joural of Pharmacy | 3 | 5 50 |
| Virginia Medical Monthly | 3 | 5 50 |
| Half-Yearly Compendium | 3 | 5 50 |
| Charleston Medical and Surgical Journal | 3 | 5 50 |
| Medical News and Library and Supplement | 3 50 | 6 00 |
| Herald of Health | 1 50 | 4 25 |
| Druggists' Circular | 1 50 | 4 25 |
| Tennessee Pharmacal Gazette | 1 50 | 4 25 |
| Journal of Materia Medica | 1 00 | 3 75 |
| Boston Journal of Chemistry | 1 00 | 3 75 |
| Baltimore Physician and Surgeon | 1 00 | 3 75 |
| Physician and Pharmacist | 1 00 | 3 75 |

